# Supplementary material for: Resistance Mechanisms of Fluoroquinolone in Escherichia coli Isolated from Taihe Black-Boned Silky Fowl Exhibiting Abnormally Slow Fluoroquinolone Metabolism in Jiangxi, China
Source: Antibiotics (Basel). 2025 Sep 21;14(9):955. doi: 10.3390/antibiotics14090955 (PMC12466724; doi:10.3390/antibiotics14090955)
Supplement: Supplementary file 1 [file antibiotics-14-00955-s001.zip › S7 FQs residues detection method.pdf]

Analytical method and validation for FQs

(With minor modification from Yuan et al., 2023. J Food Sci. 88(4): 1743-52. doi: 10.1111/1750-3841.16501)

The 1.0 g of fecal, breast meat, egg, and feed samples were accurately weighted, and then placed into 50 mL centrifuge tube, respectively.

During the extraction process for ENR, CIP, and OFX, 50 μL of 1.0 μg/mL internal standards, and 10.0 mL of 0.5% formic acid/ acetonitrile (V/V) were added into the centrifuge tube. And the mixture was vortexed for 1 min, sonicated for 20 min, and centrifuged at 4000 r/min for 5 min. Then the extract was transferred to another 50 mL centrifuge tube, and the residue was repeatedly extracted with 10.0 mL 0.5% formic acid/ acetonitrile (V/V). The extracts were combined. During the extraction process for TMP and SCP, 10.0 mL of acetonitrile was added into the centrifuge tube. During the analysis of skin, 5 g of anhydrous sodium sulfate was added. And the mixture was vortexed for 1 min, sonicated for 20 min, and centrifuged at 4000 r/min for 5 min. Then the extract was transferred to another 50 mL centrifuge tube, and the residue was repeatedly extracted with 10.0 mL acetonitrile. The extracts were combined.

For purification, 10 mL of hexane was added in the combined extracts. The extracts were shaken for 10 min, and centrifuged at 4000 r/min for 5 min. Then the upper layer was discarded. The lower layer was dried by nitrogen in 50℃ water bath. The residue was subsequently reconstituted with 2 mL of MeOH: water (1: 9, V/V). The supernatant was filtered through a 0.22-μm polytetrafluoroethene (PTFE) filter for further analysis.

Analysis was performed on an LC 30AD ultra-performance liquid chromatography (UPLC) (Shimadzu, Kyoto, Japan), coupled with a 6500 QTRAP triple-quadrupole mass spectrometry (MS/MS) (SCIEX, Framingham, MA, USA). Separation was achieved on a BEH C18 HPLC column (2.1 × 100 mm, 1.7 μm) (Waters, Milford, MA, USA) at 40℃. Mobile phases were methanol (A), and water with 0.1% formic acid (B), with a flow rate of 0.3 mL/min. The gradient elution program performed as follows:

0-0.01 min: 10% B; 0.01-3 min 40% B; 3-5 min 100% B; 5-5.1 min 10% B; 5.1-6 min 10% B. The total cycle time was 6 min. The injection volume was 5 μL. For the MS/MS system, a positive electrospray ionization source mode was used. Ion-spray voltage was 4500 V, ion source temperature was 500°C, gas 1 and gas 2 pressure were both 50 psi, curtain gas pressure was 40 psi. The multiple reaction monitor (MRM) mode was applied to each analyte, with a dwell time of 3 ms. The MRM transition parameters for the three analytes and three internal standards are listed in Table A1.

For samples containing high concentrations of antibiotics, the concentration of the extract exceeded the linear range of the standard curve, necessitating appropriate dilution prior to analysis. The internal standard method was employed for the quantitative determination of ENR, CIP, and OFX.

The method was validated on blank fecal, breast meat, egg, and feed samples of Taihe Black-Boned Silky Fowls (Table A2). The matrix-matched calibration curves ranged from 1 to 200 μg/L (correlation coefficients (R^2) > 0.99). The limit of detection (LOD) was defined as S/N ≥ 3, which was 0.3 μg/kg. The limit of quantification (LOQ) was defined as S/N ≥ 10, which was 1.0 μg/kg. The average recoveries of ENR, CIP, and OFX at the spiked levels of 10 μg/kg with six replicates for each level were 78.9%-106.2%. The intra-day and inter-day RSDs for the spiked mixture samples performed on the same day and six consecutive days in duplicate (n = 6) were 4.8%-9.1% and 4.6%-8.8%, respectively.

Table S7 Three standards, and internal standards and their multi-reaction monitoring parameters.

Compound	Abbr.	Parent ion (m/z)	Daughter ion (m/z)	Collision energy (eV)
enrofloxacin	ENR	360	342 [a]	20
			316	20
ciprofloxacin	CIP	332	314 [a]	20
			231	42
ofloxacin	OFX	362	318.1[a]	15
			261.1	26
d_3-ofloxacin	d_3-OFX	365.1	321.1[a]	20
			261	30
d_5- enrofloxacin	d_5-ENR	365	347 [a]	25
			321	20
d_8- ciprofloxacin	d_8-CIP	340	322 [a]	25
			296	20

[a] quantifier ion

Table S8 Analytical method validation parameters: LODs, LOQs, recoveries of target compounds, and RSDs (n = 6).

Analytes	LOD (μg/kg)	LOQ (μg/kg)	Recovery (RSD [a], RSD [b]) (%)			
			Breast meat	Fecal	Egg	Feed
ENR	0.3	1.0	78.9 (7.3, 5.4)	103.9 (6.9, 7.1)	94.4 (6.6, 5.8)	96.7 (9.1, 4.6)
CIP	0.3	1.0	87.2 (6.4, 7.7)	97.0 (6.5,7.2)	89.9 (4.8, 7.2)	88.7 (8.8, 8.0)
OFX	0.3	1.0	83.9 (7.5, 8.8)	89.9 (5.4, 4.6)	106.2 (7.6, 6.3)	90.9 (7.5, 8.6)

[a] the intra-day relative standard deviations (n = 6)

[b] the inter-day relative standard deviations (n = 6)
