# Supplementary figures and images for: Resistance Mechanisms of Fluoroquinolone in Escherichia coli Isolated from Taihe Black-Boned Silky Fowl Exhibiting Abnormally Slow Fluoroquinolone Metabolism in Jiangxi, China
Source: Antibiotics (Basel). 2025 Sep 21;14(9):955. doi: 10.3390/antibiotics14090955 (PMC12466724; doi:10.3390/antibiotics14090955)

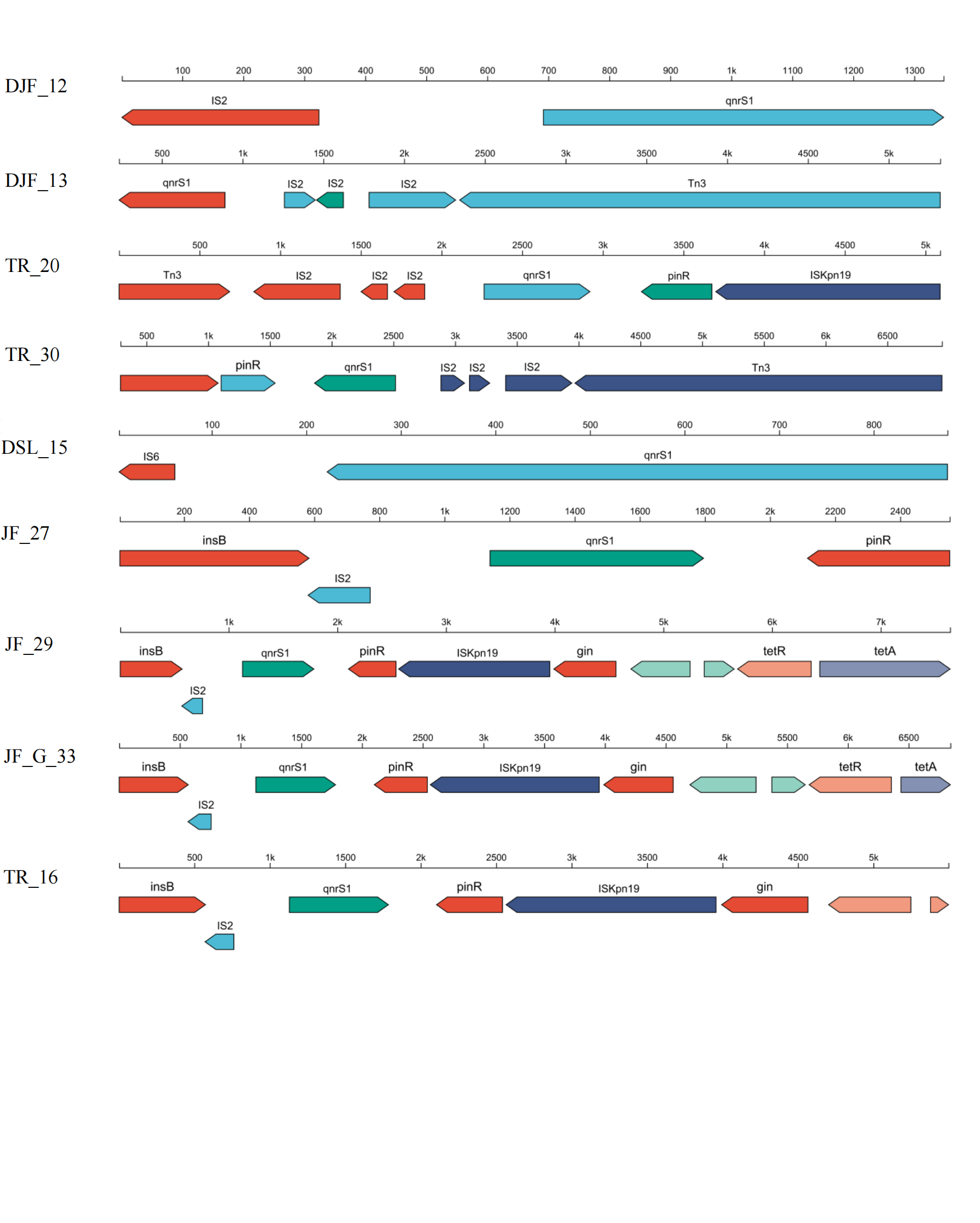

Supplement: Supplementary file 1 [file antibiotics-14-00955-s001.zip › S1 Fig. The genetic environment of qnrS1 in E. coli isolates_20250915.tif]
